# A pathologically confirmed case of combined amyotrophic lateral sclerosis with *C9orf72* mutation and multiple system atrophy

**DOI:** 10.1111/neup.12808

**Published:** 2022-06-23

**Authors:** Andrew King, Yuan Kai Lee, Shalmai Jones, Claire Troakes

**Affiliations:** ^1^ Department Of Clinical Neuropathology King's College Hospital, Denmark Hill London UK; ^2^ London Neurodegenerative Diseases Brain Bank and Department of Basic and Clinical Neuroscience, Institute of Psychiatry, Psychology and Neuroscience, King's College London London UK; ^3^ Department of Neurology Eastbourne District General Hospital, King's Drive East Sussex UK

**Keywords:** amyotrophic lateral sclerosis, C9orf72, frontotemporal lobar degeneration, multiple system atrophy, parkinsonian

## Abstract

Hexanucleotide repeat expansions in *C9orf72* account for a large proportion of cases of amyotrophic lateral sclerosis (ALS) and frontotemporal lobar degeneration. There have been occasional reported cases associated with this expansion but also additional extrapyramidal clinical features. However, only very rarely has there been pathological confirmation of a parkinsonian syndrome associated with a *C9orf72* repeat expansion. To date, as far as we are aware, there have been no reported pathologically confirmed cases of ALS with *C9orf72* mutation and multiple system atrophy (MSA). We report a case of a man who initially presented with extrapyramidal features, including cogwheel rigidity, and, therefore, was clinically considered likely to have Parkinson's disease or a parkinsonian syndrome. Subsequent examination six months later revealed additional abnormal upper and lower motor neuron signs, raising the strong possibility of ALS. He had a rapid clinical decline and died 16 months after the first presentation. It was noted that his father also had ALS, and that his mother had a parkinsonian syndrome, suggestive of progressive supranuclear palsy. The macroscopic and microscopic examination of the brain and spinal cord revealed ALS pathology with neuronal loss, especially of the anterior horns of the cord and the motor cortex. This was associated with numerous neuronal cytoplasmic inclusions immunoreactive for phosphorylated transactivation response DNA‐binding protein of 43 Da (TDP43). There were additional pathological features, including p62‐immunoreactive cerebellar neuronal cytoplasmic inclusions, fully in keeping with a *C9orf72* repeat expansion, and this was confirmed on molecular analysis. However, there was also α‐synuclein pathology in the form of oligodendroglial cytoplasmic inclusions in the basal ganglia, cerebellum, and brainstem, indicative of MSA. To our knowledge, this is the first reported case of pathologically confirmed combined ALS‐*C9orf72* and MSA.

## INTRODUCTION

Expansions of the hexanucleotide repeat GGGGCC in the *C9orf72* gene (C9orf72) have been identified as a major cause of familial and sporadic amyotrophic lateral sclerosis (ALS) and frontotemporal lobar degeneration (FTLD).^1^, ^2^ Following this discovery, there has been considerable interest as to whether the repeat expansions are associated with other neurodegenerative disorders, including atypical parkinsonian disorders such as progressive supranuclear palsy (PSP), corticobasal degeneration (CBD), and multiple system atrophy (MSA). Large studies on such cases have found the repeat expansion only rarely or not, to our knowledge, in any pathologically confirmed cases of MSA.^3^, ^4^ Here, we report a case of a man who first presented clinically with extrapyramidal signs but later exhibited both upper and lower motor neuron signs. Neuropathologically, he was proved to have both MSA and ALS associated with *C9orf72* repeat expansion type pathology. This to our knowledge is the first report of a pathologically proven case with this combination of diseases.

## CLINICAL SUMMARY

A 60‐year‐old man first presented with a history of falls. On examination, the patient showed prominent extrapyramidal features with cogwheel rigidity and reduced pendulum of the right arm as well as slight torticollis and negative glabellar sign. There was no evidence of postural instability or cerebellar signs. A subsequent magnetic resonance imaging provided unremarkable results, because there was no hallmark “hot cross bun” sign in the pons on T2‐weighted images and no hyperintense signal of the putaminal rim. In addition, no cerebellar atrophy was noted. Dopamine transporter, so‐called DaT scan, revealed features consistent with loss of presynaptic dopaminergic terminals. This was supportive of a parkinsonian disorder, including Parkinson's disease (PD). Subsequent neuropsychological assessment only exposed mild behavioral changes with some attentive and visual memory problems. Further clinical examination six months later, however, revealed upper and lower motor neuron signs characterized by tongue fasciculation, wasting of intrinsic muscles, and spasticity and hyper‐reflexia of the upper limbs. The lower limbs showed bilateral extensor plantor response and increased muscle tone. Again, the patient showed none of the cerebellar signs, postural instability, or autonomic instability. There was no evidence of further cognitive decline, including frontotemporal dementia. Electromyography revealed marked denervation of striated muscles in bulbar, cervical, thoracic, and lumbar segments. Based on these observations, the patient was diagnosed as having ALS. He had previously been prescribed levodopa (L‐dopa), to which he had a poor response. Given the amended diagnosis, the patient's treatment was subsequently changed to riluzole. The patient's father had had ALS and had died within one to two years after the diagnosis. There was also a suggestion that his mother had had a parkinsonian disorder, possibly PSP, and died five to six years after the diagnosis. The patient had a brother who had had no known neurological abnormalities. However, no other familial information was available. The patient died 16 months after the first presentation, with the cause of death being stated as ALS. A limited postmortem was performed.

## PATHOLOGICAL FINDINGS

**Fig 1:**
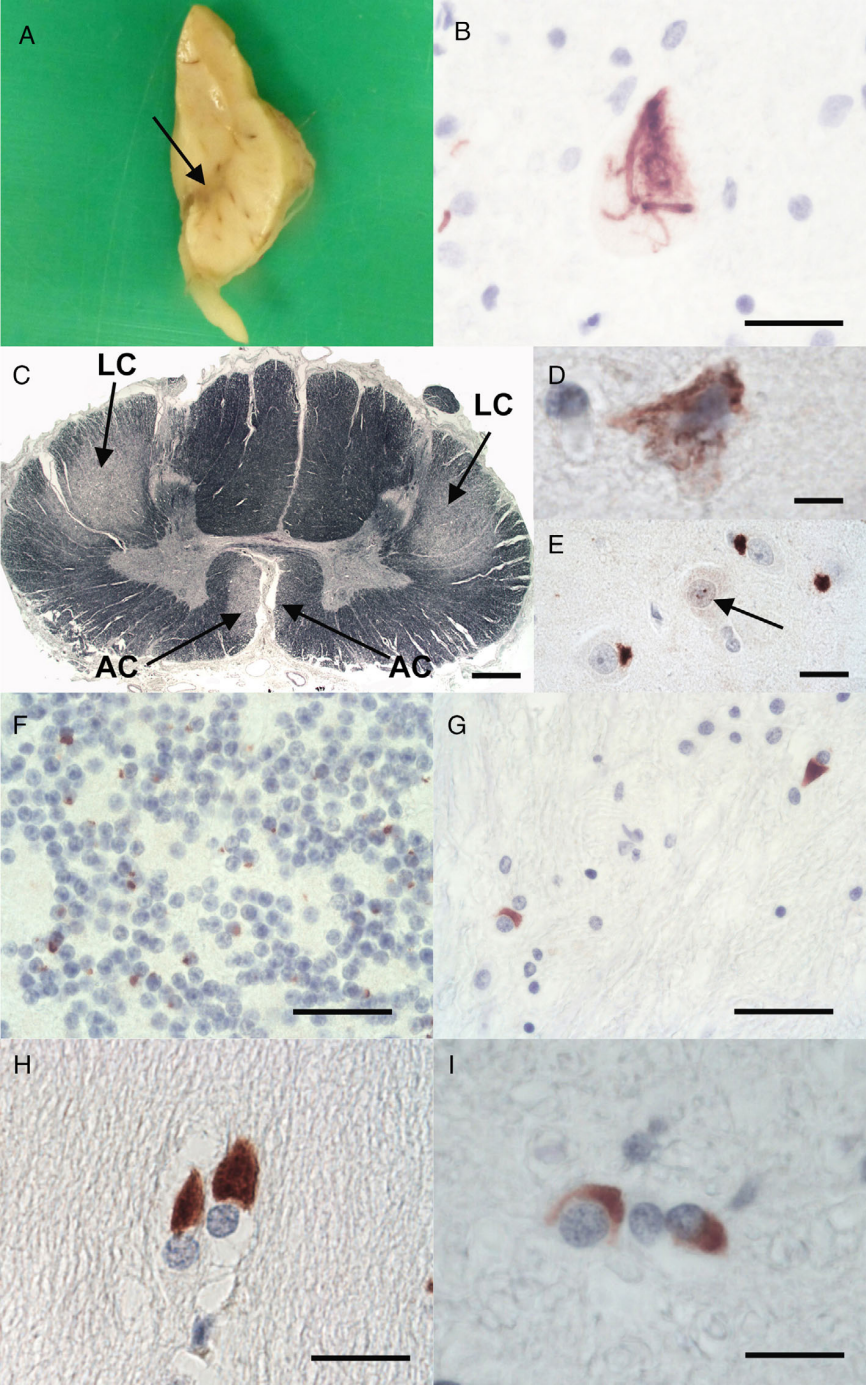
Macroscopic (A), semimacroscopic (C), and microscopic (B, D–I) findings of the brain and spinal cord. (A) An axial slice of the right half of the midbrain shows depigmentation of the substantia nigra (arrow). (B) A lower motor neuron containing a p‐TDP43‐immunoreactive skein‐like inclusion is observed in the spinal cord anterior horn. (C) KB staining highlights myelin pallor of the lateral (LC) and anterior (AC) corticospinal tracts, indicative of upper motor neuron damage. (D) A p‐TDP43‐immunoreactive NCI is observed in an upper motor neuron of the motor cortex. (E) In the hippocampal CA4 sector, “starburst‐like” p62‐immunoreactive NCIs and an NII (arrow) are seen. (F) There are numerous p62 immunoreactive NCIs (F) in the cerebellar granule cell layer. (G‐I) Moderate‐to‐abundant α‐synuclein‐immunoreactive GCIs are observed in the cerebellar white matter (G), pontine transverse fibers (H), and the grey matter of the spinal cord (I). Scale Bars: 50 μm (B), 1100 μm (C), 30 μm (D), 40 μm (E), 100 μm (F, G), 30 μm (H), 20 μm (I).

The right half of the brain and some spinal cord segments were fixed in 10% buffered formalin. The left half of the brain and other spinal cord segments were frozen. The fixed right cerebral hemisphere weighed 709 g, and the right brainstem and cerebellum weighed 93 g. Macroscopic examination revealed mild cerebral atrophy with some moderate widening of the lateral ventricle. The basal ganglia, thalamus, and hippocampus appeared normal. Pigment was lost in the substantia nigra (Fig. 1A) and much less in the locus ceruleus. Neither the brainstem nor the cerebellum showed atrophy, whereas the spinal cord showed atrophy of the ventral nerve roots.

Microscopic samples were taken from the cerebrum, brainstem, cerebellum, and spinal cord. Formalin‐fixed, paraffin‐embedded sections were stained with hematoxylin and eosin (HE). Sections from the spinal cord and motor cortex were also stained with Luxol fast blue/cresyl violet, so‐called Klüver‐Barrera (KB). Section thickness was 14 μm for KB staining and 7 μm for other staining. On the relevant sections, immunohistochemistry was performed with primary antibodies against hyperphosphorylated tau (p‐tau) (mouse monoclonal, clone AT‐8; Autogen Bioclear UK, Wiltshire, UK; 1:500), p62 (mouse monoclonal, BD Biosciences, Erembodegem, Belgium; 1:100), α‐synuclein (mouse monoclonal, clone 42/α‐synuclein; Novocastra Laboratories, Newcastle upon Tyne, UK; 1:500), amyloid‐β (Aβ) (mouse monoclonal, clone 4G8; Chemicon, Temecula, CA, USA; 1:12000), transactivation response DNA‐binding protein of 43 kDa phosphorylated at serine residues 409 and 410 (p‐TDP43) (rabbit polyclonal, Cat. No. S409/410‐2; Cosmo Bio, Tokyo, Japan; 1:1500), and glial fibrillary acidic protein (GFAP) (rabbit polyclonal, Cat. No. Z0334; Dako, Glostrup, Denmark; 1:1000). Sections were deparaffinized, rehydrated, and rinsed in phosphate‐buffered saline (PBS), pH 7.6and quenched for endogenous peroxidase activity. Leica BONDMAX (Leica Biosystems, Wetzlar, Germany) epitope retrieval sets were used for p‐TDP43 (ER1 for 30 min), tau (ER1 for 20 min), GFAP (ER1 for 5 min), and p62 (ER2 for 20 min). For both α‐synuclein and Aβ, 80% formic acid pretreatment for 1 h was used. After incubation with the primary antibodies followed by rinsing phosphate‐buffered saline (PBS), pH 7.6, sections were incubated with appropriate biotinylated secondary antibodies (Dako), followed by the avidin‐biotin‐enzyme complex using a Vectastain Elite ABC Kit (Vector Laboratories, Peterborough, UK). Finally, sections were incubated for 5–10 min with 0.5 mg/mL 3,3′‐diaminobenzidine (Sigma‐Aldrich, Dorset, UK) as the chromogen in Tris‐buffered saline (TBS), pH 7.6, containing 0.05% H_2_O_2_. Cell nuclei were counterstained with Harris' alum hematoxylin. The immunohistochemical findings are summarised in Table 1. To search for possible co‐localisation of p‐TDP43 and α‐synuclein, double immunofluorescence staining was performed on the putamen and amygdala. Sections were subsequently pretreated by immersion in formic acid for 5 min before microwaving in citrate buffer and then processed for 45 min blocking nonspecific antibody binding using nonimmune goat serm in PBS at a dilution of 1:10. Sections were incubated for 1 h at 37°C with primary antibodies against α‐synuclein (mouse monoclonal, clone 42/α‐synuclein; BD Biosciences, Erembodegem, Belgium; 1:500) and p‐TDP43 (rabbit polyclonal, Cat. No. pS409/10; Cosmo, Tokyo, Japan; 1:2000). After rinsing in PBS, sections were incubated for 45 min under dark conditions with Alexa Fluor 568‐conjugated goat anti‐mouse IgG (Invitrogen, Paisley, UK) Alexa Fluor 488‐conjugated goat anti‐rabbit IgG (Invitrogen). Autofluorescence was quenched by incubating the sections in Sudan Black for 10 min followed by eight times 3 min rinsing in PBS before coverslip mounting using Vectashield hard set media with 4′,6‐diamidino‐2‐phenylindole (DAPI). Immunoreaction was visualized using a fluorescence microscope (Zeiss AxioImager Z1, Goettingen, Germany), and images were captured using AxioVision Rel 4.8.2. (Zeiss).

**Table 1:** Immunohistochemical distribution of p‐TDP43, p62, and α‐synuclein in the patient's brain and spinal cord

Location	p62	p‐TDP43	α‐synuclein
Spinal cord anterior horn	+++ (NCI) +++ (GCI)	+++/++ (NCI) ++ (GCI)	++ (GCI)
Spinal cord white matter	+++ (GCI)	+ (GCI)	++/+ (GCI)
Medulla oblongata XII	+++/++ (NCI) +++ (GCI)	+++/++ (NCI) ++ (GCI)	−
Medulla oblongata Ret/Pyr	+++/++ (GCI‐Ret) ++ (GCI‐pyr)	+ (GCI‐Ret) ‐ (GCI‐pyr)	+++/++ (GCI‐Ret) + (GCI‐pyr)
Pontine nuclei	++ (GCI)	−	++ (GCI)
Pontine transverse fibers	+++ (GCI)	−	+++ (GCI)
Midbrain substantia nigra	++ (NCI) +++ (GCI)	++ (NCI) ++ (GCI)	++ (GCI)
Midbrain cerebral peduncle	+++ (GCI)	++ (GCI)	+++ (GCI)
Motor cortex	+++ (NCI) ++ (GCI)	+ (NCI) ++ (GCI)	NA
Hippocampus CA1‐CA4	+++ (NCI‐star) + (GCI) ++ (NII)	−	−
Hippocampus dentate gyrus	+++ (NCI)	−	−
Amygdala	+++ (NCI) +++ (GCI)	+(NCI) + (GCI)	+ (GCI)
Frontal cortex	+++ (NCI) ++ (GCI)	−	++/+ (GCI)
Frontal white matter	++ (GCI)	−	++ (GCI)
Temporal cortex	+++ (NCI) ++ (GCI)	−	−
Temporal white matter	+ (GCI)	−	+ (GCI)
Putamen	++ (NCI) +++ (GCI)	+ (NCI) + (GCI)	++ (GCI)
Cerebellum granule layer	+++/++ (NCI)	−	−
Cerebellum molecular layer	+++(NCI)	−	−
Cerebellum white matter	+++ (GCI)	−	++ (GCI)

Number of inclusions are scored as negative (−), sparse (+), moderate (++), and frequent (+++). GCI, glial cytoplasmic inclusion; NCI, neuronal cytoplasmic inclusion; NII, neuronal intranuclear inclusion; NA, not applicable; Pyr, pyramid; Ret, reticular formation; star, starburst‐like inclusions; XII, hypoglossal nerve nucleus.

**Fig 2:**
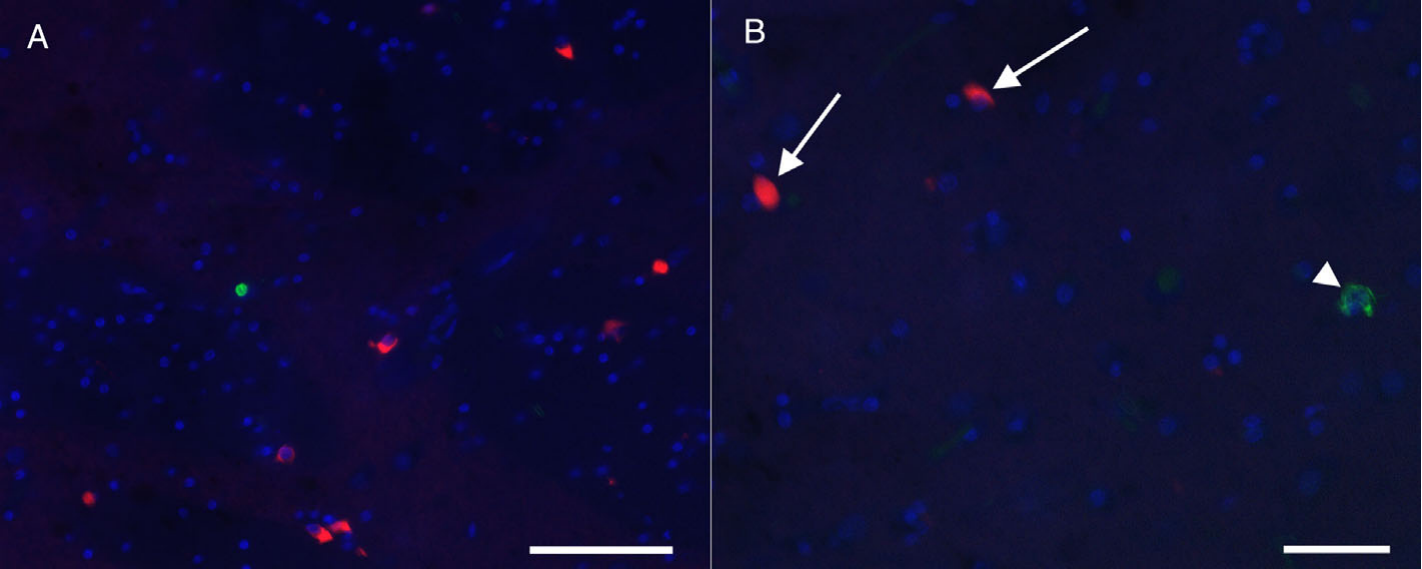
Microscopic double immunofluorescence findings of the putamen. (A, B) Arrowheads and arrows indicate NCIs and GCIs, respectively. Numerous α‐synuclein‐immunoreactive GCIs (red) and occasional p‐TDP43‐immunoreactive GCIs and NCIs (green) are found. There is no evidence of colocalization of α‐synuclein and p‐TDP43. Scale bars: 100 μm (A), 50 μm (B).

The spinal cord sections showed neuronal loss and gliosis of the anterior horn, including a surviving neuron containing Bunina bodies. The motor cortex and hypoglossal nerve nuclei showed mild‐to‐moderate neuronal loss and gliosis. The substantia nigra and the basal ganglia, particular the putamen, showed moderate neuronal loss and mild gliosis. By contrast, other regions, including the pontine nuclei and inferior olivary nuclei, appeared preserved. No significant gliosis was evident in the transverse pontine fibers. Both the Purkinje cell layer and granule cell layer of the cerebellum showed good preservation of the neurons. However, the cerebellar white matter, including the middle cerebellar peduncles, showed mild gliosis. In the spinal cord, there were p‐TDP43‐immunoreactive abnormal structures, such as neuronal cytoplasmic inclusions (NCIs), including skein‐like inclusions (Fig. 1B), and glial cytoplasmic inclusions (GCIs). KB staining revealed myelin pallor of the lateral and anterior corticospinal tracts (Fig. 1C) in the spinal cord. Neuronal loss and appearance of p‐TDP43‐immunoreactive NCIs and GCIs were observed in the motor cortex (Fig. 1D), hypoglossal nerve nuclei, substantia nigra, and basal ganglia. The p‐TDP43‐immunoreactive NCIs were detectable in the amygdala but undetectable in the hippocampus, neocortex, and cerebellum. On p62 immunohistochemistry, there were numerous p62‐positive NCIs and GCIs in the neocortex, brainstem, basal ganglia, amygdala, hippocampus, cerebellum, and spinal cord. Many of these NCIs appeared dot‐like. In the hippocampus, these findings were particularly evident in the Cornu Ammonis 4 (CA4) sector, with a “starburst”‐like pattern (Fig. 1E), and associated with occasional neuronal intranuclear inclusions (NIIs) (Fig. 1E). In the cerebellum, there were numerous p62‐immunoreactive NCIs in the granule cell layer (Fig. 1F) and molecular layer. Immunohistochemically, these were negative for p‐TDP43. Together, the combination of the p‐TDP43 pathology and numerous p62‐positive, p‐TDP43‐negative inclusions was in keeping with ALS associated with a *C9orf72* repeat expansion. In addition, immunohistochemistry for α‐synuclein revealed several immunoreactive GCIs in the basal ganglia, especially the putamen, cerebellar white matter (Fig. 1G), midbrain, pons (Fig. 1H), medulla oblongata, and spinal cord (Fig. 1I). The intermediolateral columns of the spinal cord exhibited occasional appearance of α‐synuclein‐immunoreactive GCIs. In addition, occasional α‐synuclein‐immunoreactive GCIs were observed in the cerebral white matter and amygdala. These GCIs appeared to be predominantly localized in oligodendrocytes, and many appeared to be cup‐shaped; the overall pattern was entirely in keeping with the pathology of MSA. No Lewy bodies were seen. Double immunofluorescence for p‐TDP43 and α‐synuclein revealed a separate localization of immunoreactivities for α‐synuclein and p‐TDP43 GCIs in the putamen (Fig. 2A) and the occasional presence of p‐TDP43‐immunoreactive NCIs (Fig. 2B). The immunoreactivities for these proteins were not colocalized. Similarly, within the amygdala, there were sparser p‐TDP43‐immunoreactive NCIs and α‐synuclein‐immunoreactive GCIs and these were not colocalized. The Alzheimer's disease pathology neurofibrillary Braak stage was 0, and the Thal amyloid phase was 1. Repeat‐primed poymerase chain rection (PCR) of frozen cerebellar tissue samples was performed and revealed a heterozygous pathogenic GGGGCC expansion in *C9orf72* greater than 60 repeats (Sheffield Diagnostic Genetic Service, Sheffield Children's NHS Foundation Trust (Sheffield, UK)).

## DISCUSSION

The *C9orf72* repeat expansion is the most common mutation associated with familial ALS and the familial FTLD‐ALS spectrum, including ALS, ALS‐dementia complex, ALS‐FTLD, and FTLD. Neuropathologically, there are characteristic p62‐positive, p‐TDP43‐negative inclusions in the brain, most notably in the cerebellum and hippocampus.^5^ In addition, the classical p‐TDP43 pathology is associated with the FTLD‐ALS spectrum. Occasionally within this spectrum, patients have been reported clinically to have extrapyramidal signs and symptoms, and raises a question of whether *C9orf72* repeat expansions could be associated with parkinsonian disorders such as PD, dementia with Lewy bodies, PSP, CBD, and MSA. On testing clinically diagnosed parkinsonian patients for *C9orf72* repeat expansions, Lesage *et al*. found that of 1446 subjects, including 25 with clinically diagnosed MSA, five carried the repeat expansion, including three patients with PD, one with CBD, and one with PSP.^3^ Similarly, in a Chinese population of 1000 patients with parkinsonism, including 381 with clinically diagnosed MSA, none showed the expansion.^6^ In a mixed cohort of clinically and pathologically diagnosed cases of atypical parkinsonism, analysis for the repeat expansion revealed one case of clinical PSP with an intermediate repeat length, and in the 291 neuropathologically confirmed cases, including 96 cases of MSA, no cases showed an expansion longer than 22 repeats.^4^ A separate study by the same group showed no pathological *C9orf72* repeat expansions in 100 pathologically confirmed cases of MSA.^7^ Goldman *et al*. described one case of clinically diagnosed mixed ALS‐MSA phenotype with evidence of a *C9orf72* repeat expansion; however, it was not pathologically confirmed.^8^, ^9^, ^10^ Therefore, to our knowledge, the present report describes the first pathologically confirmed case of characteristic ALS with *C9orf72* repeat expansion pathology together with typical MSA α‐synuclein pathology. The early parkinsonian clinical features of cogwheel rigidity and decreased arm swinging together with poor response to L‐dopa were most likely to be explained by neuronal loss, gliosis, and α‐synuclein oligodendroglial pathology in the putamen and substantia nigra, thus giving a parkinsonian subtype of MSA, also known as MSA‐P. Similarly, neuronal preservation of the Purkinje cell and granule cell layers of the cerebellum, pontine nuclei, and inferior olivary nuclei probably explained the lack of cerebellar signs, despite both the cerebellar white matter α‐synuclein pathology and the separate *C9orf72* repeat expansion‐associated p62 pathology in the granule layer. The lack of dysautonomic signs may have resulted from a relatively small number of α‐synuclein immunopositive GCIs present in the intermediolateral columns. The later appearance of the additional upper and lower motor ALS‐type clinical features, including muscle weakness, spasticity, and dysphagia was more likely caused by motor neuronal loss of the motor cortex, medullary hypoglossal nerve nucleus, and spinal cord anterior horn together with associated p‐TDP43 pathology. Interestingly, the substantia nigra contained abundant p‐TDP43, α‐synuclein, and p62 deposits, suggesting involvement in the motor signs of both the parkinsonian and ALS disease processes. Presumably, the relatively rapid nature of loss of the upper and lower motor neurons was the reason that the neurological ALS components predominated on the later clinical examinations.

There was evidence of a family history of ALS, from the father, and a possible parkinsonian syndrome, from the mother. In addition to early extrapyramidal signs, more atypical ALS‐type neurological and neurophysiological features were detected later. Although it is evident that the patient inherited the ALS‐associated *C9orf72* GGGGCC expansion from his father, the explanation of the MSA‐type clinical and pathological features is somewhat problematic. Although it would be tempting to suggest that the patient's mother actually had MSA but not PSP, there is no actual pathological evidence to confirm this. Furthermore, MSA, unlike ALS‐*C9orf72*, has been shown to only very rarely be inherited in an autosomal dominant or recessive manner.^11^, ^12^, ^13^ Genome‐wide studies have been performed recently, and there have been associations among very occasional mutations in the synuclein alpha gene (*SNCA*), copy number variants of *SNCA*, mutations in the coenzyme Q2 gene (*COQ2*), and even some mutations in the microtubule‐associated protein tau gene (*MAPT*) and the glucosylceramidase beta 1 gene (*GBA1*).^14^ Therefore, the evidence at present suggests that MSA is a sporadic disease and may be associated with rare genetic variants increasing susceptibility.

It is also possible to speculate that the *C9orf72* mutation separately inherited from the father exacerbated the effects of the MSA‐associated variants to cause MSA‐type symptoms and pathology. There is some circumstantial evidence that *C9orf72* repeat expansions may have these effects. We previously described a patient with a *C9orf72* repeat expansion and a usually non‐pathogenic *MAPT* variant that, nevertheless, presented with an FTLD pattern mainly associated with tau and p62 pathologies and less p‐TDP43 pathology.^15^ Bieniek *et al*. also showed evidence that FTLD‐*C9orf72* had an increased propensity for tau pathology compared to FTLD associated with progranulin mutations but not sporadic FTLD.^16^ Robinson and colleagues, however, found no excessive tau pathology in cases of ALS or FTLD with *C9orf72* repeat expansions.^17^ Whether *C9orf72* repeat expansions can “unmask” or “promote” otherwise less or non‐pathogenic variants of *MAPT* or *SNCA* remains an intriguing possibility.

## DISCLOSURE

The authors declare no conflicts of interest for this article.
